# Land Use Planning to Reduce Flood Risk: Opportunities, Challenges and Uncertainties in Developing Countries

**DOI:** 10.3390/s22186957

**Published:** 2022-09-14

**Authors:** Rita Der Sarkissian, Mario J. Al Sayah, Chadi Abdallah, Jean-Marc Zaninetti, Rachid Nedjai

**Affiliations:** 1 University of Gustave Eiffel, University of Paris Est Creteil, Ecole des Ingénieurs de la Ville de Paris (EIVP), LAB’URBA, F-77454 Marne-la-Vallée, France; 2 National Council for Scientific Research, Remote Sensing Center, Natural Hazard, Beirut 11-8281, Lebanon; 3 Centre D’études et de Développement des Territoires et de l’Environnement, Université d’Orléans, 45100 Orléans, France

**Keywords:** disaster risk reduction, resilience, land-use planning, geospatial, remote sensing

## Abstract

Land use planning for flood risk reduction has been significantly addressed in literature. However, a clear methodology for flood mitigation oriented land-use planning and its implementation, particularly in developing countries like Lebanon, is still missing. Knowledge on land use planning is still in its earliest stages in Lebanon. A lack of hazard-informed land use planning coupled to random land cover pattern evolution characterize the country. In response, this study focuses on the opportunities, challenges and uncertainties resulting from the integration of land use planning into efficient Disaster Risk Reduction (DRR). For this purpose, GIS-based analyses were first conducted on the current land use/land cover (LU/LC) of the Assi floodplain. Then, the areas land cover was retraced and its evolution after several flood occurrences was assessed. Subsequently, a flood hazard-informed LU/LC plan was proposed. The latter is mainly based on the spatial allocation of land-uses with respect to different flood hazard levels. This approach resulted in the production of a land use planning matrix for flood risk reduction. The matrix approach can serve as a tool for designing sustainable and resilient land cover patterns in other similar contexts while simultaneously providing robust contributions to decision-making and risk communication.

## 1. Introduction

An important aspect of Disaster Risk Reduction (DRR) is the implementation of risk-based land-use planning and regulations to reduce the underlying causes of disasters and their resulting losses [1,2,3]. The integration of DRR in urban land use planning was set as a priority action in both the Hyogo and the Sendai Frameworks for DRR [4]. By definition, land use planning is a nonstructural measure that implies cautious investments and the utilization of land and natural resources in manners that ensure sustainable development [5] and risk reduction [6,7]. Moreover, land use planning is directly responsible for the protection of infrastructure and assets [8]. Land use planning also offers opportunities for building resilience and limiting vulnerabilities and risk by controlling expansions of land cover classes into hazard prone areas [9,10,11]. Accordingly, land use planners have the responsibility to move communities forward by allocating secure lands and encouraging the construction of more hazard-resilient structures, thereby decreasing “underlying risk factors” [12], p. 6.

Floods are the most widespread natural hazard both worldwide and in the Mediterranean region [13]. Yang et al. (2018) [14] and Hounkpè et al. (2019) [15] emphasized the direct relationship between flood intensity/frequency and the dynamics of land use. In this sense, zoning ordinance is an efficient tool to be used for floodplain management through land-use planning, namely through: (i) flood level delineation and (ii) formulation/application of adequate land use and building codes to guarantee the construction of flood-resilient structures [16]. 

In addition to natural exposure factors such as positions in a floodplain, changes in land use patterns and the development of new infrastructure (e.g., businesses and housing) increase the exposure and vulnerability of assets and populations [17,18]. That is why land use zoning is considered as a fundamental input for flood risk reduction [19]. Nonetheless, this task is not easy, as it requires long-term systematic planning and contributions from various disciplines, stakeholders, and decision makers [20]. In this context, land use planning proposes/regulates the most compatible uses of lands via regulations of expansions in high flood zones and the redesigning of classes in areas of low risks [21]. However, such an approach is limited by existing hazard maps, which often include debatable risk thresholds with respect to spatial extents [18]. Per example, the U.S.A 1% floodplain footprint is smaller than the Dutch’s 0.01% reference floodplain that led to the “Plan Delta” design in the 1950s.

A cross-country analysis showed that the adoption of hazard and risk-informed land-use planning decisions is still contested, even in developed countries like France, Austria, and Switzerland [22]. Mayors, officials, and politicians often consider land-use planning as a threat to their “urban power” [23,24]. That is why hazard-informed land use planning is still contested and debated. In developing countries, the implementation of land use plans and other regulatory instruments is further complicated by several intrinsic factors, namely: the presence of informal settlement, fuzzy land tenure, controversial land ownership, lack of data, resources, and capacities [25]. In this context, Lebanon generally lacks specific flood-related policies, strategies, and plans. Although Al-Assi River (Baalbek-Hermel Governorate) has flooded several times in the past (1987, 2003, 2014, 2017, and 2019), no zoning ordinance exists [26]. In addition, knowledge of land use planning is still in its earliest stages in Lebanon as the country suffers from a lack of restraints and unregulated expansions [27].

In response to this situation, this paper studies the opportunities, challenges, and uncertainties that come with land-use planning in a developing country like Lebanon. To do so, the paper examines the evolution of land cover/uses in Al-Assi floodplain and proposes strategies for reducing flood risks. For this purpose, four LU/LC time series (from 1998 to 2018) were built from various satellite imageries. The analysis of changes highlighted elements at risk in the floodplain. Finally, through a GIS framework, a LU/LC planning matrix was built. The methods presented, although applied to the Assi river, can be easily replicated in other Mediterranean watersheds. Accordingly, this approach can be considered as a contribution to flood-risk mitigation/adaptation efforts in developing Mediterranean countries.

## 2. Materials and Methods

### 2.1. Study Area

**Figure 1:**
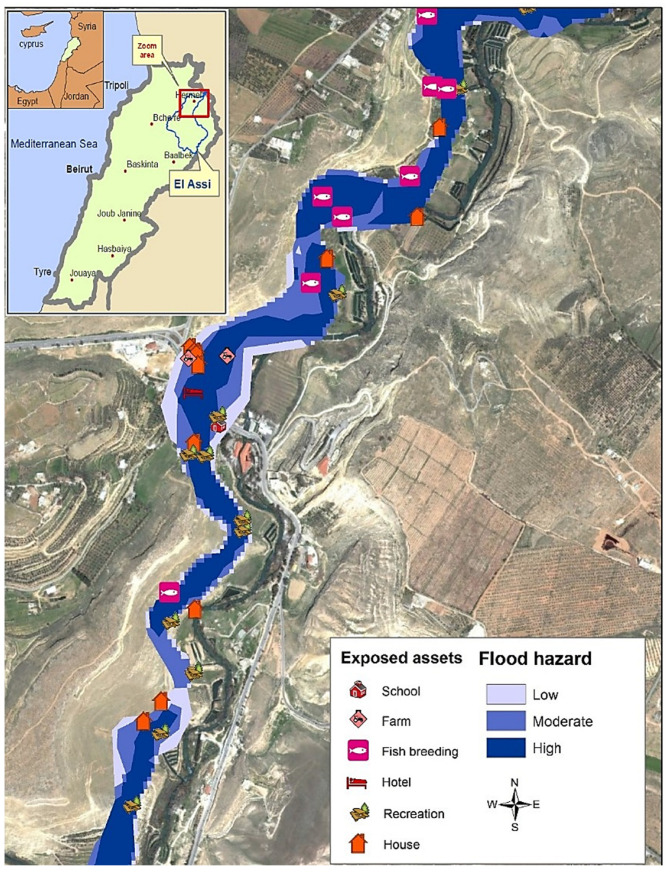
Study area of Al-Assi floodplain, Lebanon.

Al-Assi (Orontes) is a 487 km long river originating from Lebanon and extending into Syria and Turkey, respectively. The Orontes basin extends over an area of around 26,530 km^2^ and is considered as one of the largest Lebanese hydrological units [28]. The Lebanese share of the Orontes, locally named Al-Assi, is about 8% (Figure 1). It is mainly concentrated in the Baalbek-Hermel and Bekaa governorates that together form the Al-Assi catchment with an area of 350 km^2^. Although the prevailing climate is semi-arid to arid, with annual precipitations less than 400 mm [29], the basin experiences extreme seasonal variations and considerable intra-annual rainfall variability. The period extending from October to March accounts for 75–80% of total precipitations. The remainder 20–25% correspond to thunderstorms in autumn and spring flash-floods. The latter are major outbursts driving forces [30]. According to Darwish et al. (2012) [31], the region is considered as a desertification hotspot (high and very high categories) with various forms of land degradation. The area covered by the Assi has witnessed many floods in the past: 1987, 2003, 2014, 2017, and 2019. A number of human settlements have haphazardly scattered throughout the basin, sheltering about 179,000 residents and raising the population density to 85 inhabitants per km^2^ [32]. An important number of these settlements is located within the Assi floodplain, hence the high number of assets and human lives at risk. 

### 2.2. Dataset and Methodology

**Figure 2:**
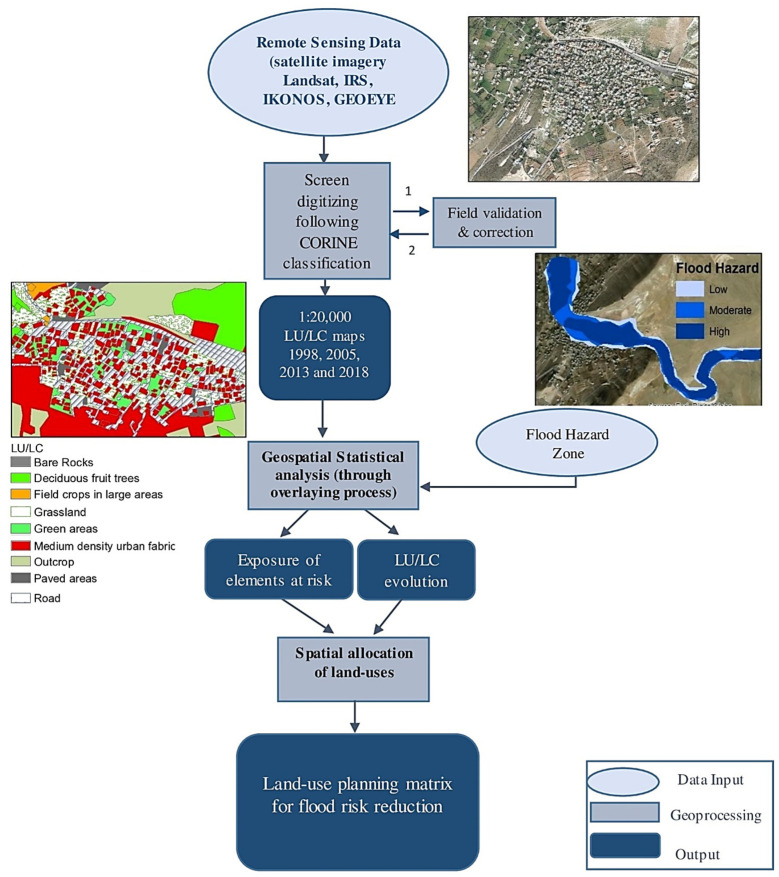
Methodological workflow diagram.

Figure 2 describes the steps of proposed methodology. First, the LU/LC maps were digitized from satellite imageries with different acquisition dates. Then, detailed time series analysis was performed to follow the development of LU/LC patterns in the floodplain. The land cover change analysis was performed through geospatial tracking of categorical changes via a polygon by polygon approach. The latter was performed using the Intersect Tool of ArcGIS (ESRI, 380 New York St, Redlands, CA, USA), where each year of the 1998–2018 time series was compared with the following. That way, each polygon from year X was compared to itself in year X + *n*, hence revealing changes. Statistics were then extracted to highlight the number of assets (structures, roads, agricultural lands, etc.) at risk, as well as their exposure levels. Finally, a hazard-informed land use plan was proposed. The latter helped develop a matrix that highlighted the different vulnerabilities of various land covers to the flood hazard. The multifunctional land use approach [33] was used for demarcating zones by level of flood hazard (low, moderate, and high) and associating them to appropriate, secure, and hazard compatible land covers.

The flood hazard zonation used in this study is based on the Flood Hazard Assessment and Mapping for Lebanon conducted in 2013 by the National Center for Remote Sensing (NCRS). The worst possible flood scenario was considered, despite its low 0.01 exceedance probability (or return period T = 100 years). The logic behind this approach is that the worst-case scenario provides a clear indication of the maximum area that will be affected by the floods. Accordingly, by adopting this scenario, the less severe ones are covered. Given the importance of data quality and its subsequent effects on outputs, high resolution multispectral satellite imagery was used for LU/LC mapping (Table 1). The Lebanese-adapted CORINE classification was used to establish the land cover maps.

LU/LC maps for the years 1998, 2005, 2013, and 2018 were obtained from onscreen digitizing of the satellite imageries presented in Table 1. Field validation was performed using the ESRI collector, an application installed on tablets to which the LU/LC shapefiles are input, hence allowing the collection and update of information by providing offline availability and syncing capacity when connected. This approach permits proper data collection with ensured spatial accuracy through the possibility of correcting polygons under question using a GPS record on field, and by capturing geolocalized coordinated photos. A total of 1627 polygons were obtained, and each was verified through a polygon by polygon approach. Areas were then computed using the field calculator function. The utilized Digital Elevation Model (DEM), with a resolution of 20-cm DEM, was obtained from drone imagery acquired bu the NCRS. More than 5000 raw images were captured from several drone photography missions over the whole floodplain. These raw images were acquired in strips, with adjoining photographs having an overlap of 80% in the flight direction and 70% between parallel flight tracks. To generate the 20 cm-resolution DEM, the images were integrated and processed in the Pix4DMapper software (Pix4D S.A. Route de Renens 24, Prilly, Switzerland) following the criteria listed by the American Society of Photogrammetry and Remote Sensing [34].

## 3. Results and Discussion

### 3.1. Land Cover Dynamics and Position with Respect to Flood Levels

**Figure 3:**
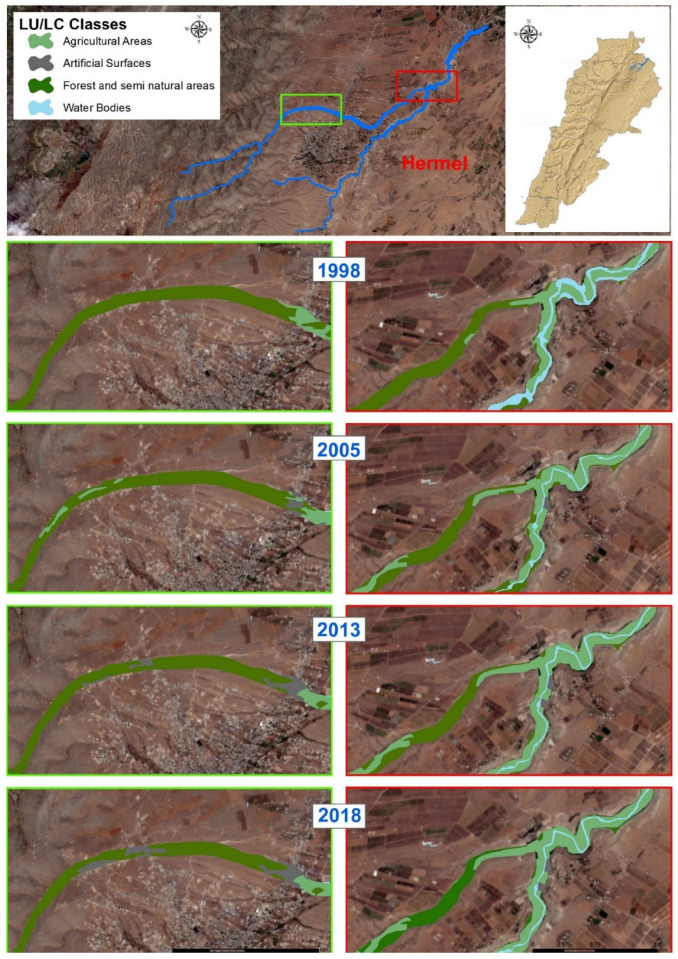
Al-Assi floodplain LULC maps for the years 1998, 2003, 2013, and 2018.

The LU/LC maps for years 1998, 2003, 2013, and 2018 are represented in Figure 3.

The analysis of the LU/LC maps revealed important changes over the years. 

**Figure 4:**
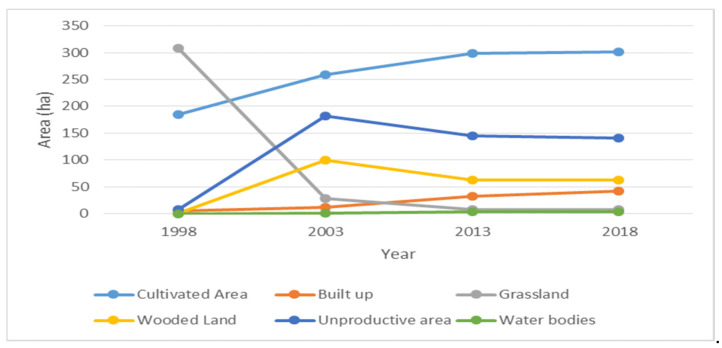
Temporal pattern of Land Use/Land Cover change.

Natural classes responsible for flood mitigation, i.e., forests, scrublands, and especially grass (Table 2 and Figure 4), have considerably decreased in the study period. These collectively presented 61.19% of the study area in 1998 and have decreased progressively to become 10.41%. Such a sharp decrease indicates a considerable regression of natural categories (approximately six times less) of protective cover during floods. In addition to the previously cited numbers, an increase in percentages (+20%) (Table 2) of unproductive lands further aggravates the deteriorating state of land-use planning in the floodplain through reduced water infiltration and amplified runoff. Accordingly, the vulnerability and exposure of populations and assets at risk have increased proportionally.

The analysis of LU/LC dynamics in Al-Assi Floodplain showed that, despite the 2003, 2014, and 2017 floods, low and medium density urban fabrics have increased by 3.4% (1998–2018), with a rate of approximately +0.2% per year. This finding is alarming since the evolution of urban cover is still increasing, despite its critical position in the floodplain. In turn, this increases the exposure of assets and populations at risk. 

In addition, another preoccupying observation was found: urban sprawls have also increased through the period 1998–2018 from 0% to 2.3%, with a rate of +0.1% per year. This further shows an intensification of urbanism in an area prone to danger. In a similar manner, an increase of 0.014% per year was observed for touristic resorts in the floodplain. In further details, a total of two hotels, two industries, one school, one villa, 44 resorts, 133 single houses, and four informal settlements were detected in the floodplain. Generally, a rate of +6.15% of urban cover was observed throughout 1998–2018 (all urban covers included). 

As can be seen from Table 3, an intersection between the 2018 LU/LC and the flood hazard map was performed. Results show that many classes fall in the high level areas. Such is the case of most urban classes. 

**Figure 5:**
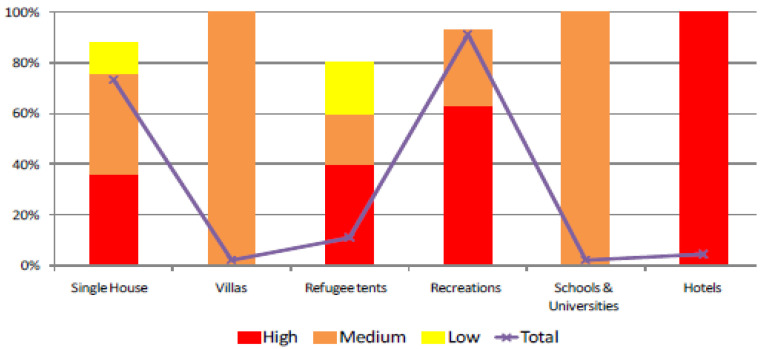
Endangered structures in various flood hazard zones.

**Figure 6:**
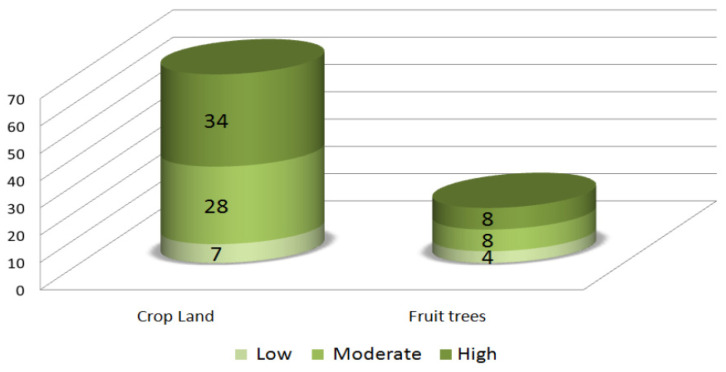
Distribution of agricultural lands (Ha) in flood hazard zones.

Following the analysis presented in Table 3, the exposure of assets was studied. Figure 5 and Figure 6 show the exposure of artificial structures and agricultural lands, respectively. It is noteworthy that all hotels present in the floodplain are situated in high-flood hazard zones. As for the only school in the floodplain, it is moderately exposed to floods. In terms of engineering material and building principles, the majority of these structures do not follow any building codes and are not flood-resistant.

### 3.2. Hazard-Informed Land Use Planning Matrix

The results presented in the previous section reflect the lack of oversight and systematic monitoring of hazard assessments into land use planning. These findings indicate the necessity of zoning ordinance is the Al-Assi floodplain. The proposed zoning ordinance is based on the transposition of information from flood hazard assessment into land-use planning to:

- Benefit as much as possible from waterfront touristic, economic, and recreational activities, along with the services offered by the ecosystem.
- Protect people and properties by following three basic principles: (a) Safe location, (b) Safe construction, and (c) Safe activities (landscaping and design of drainage and natural flood retention zones).

Special attention was given to the efforts of combining flood mitigation measures with agro-environmental practices, since the studied floodplain is situated in a rural region. 

**Figure 7:**
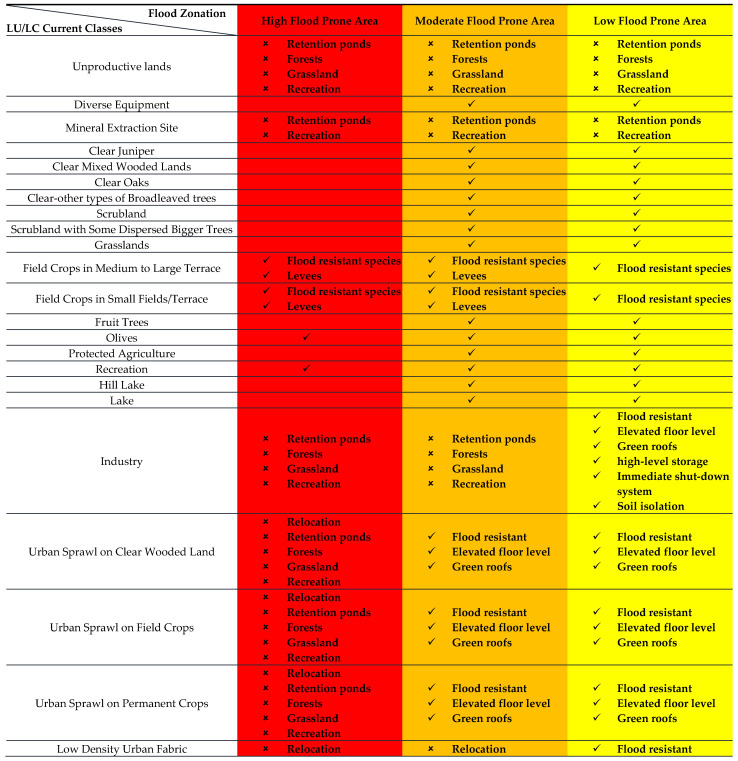

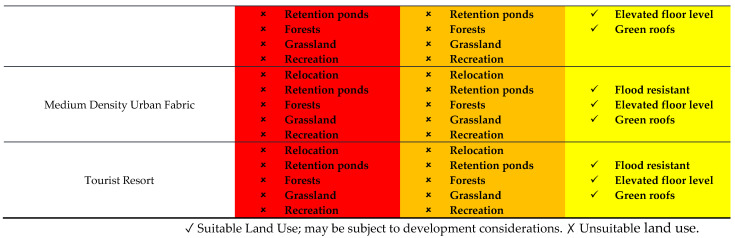
Land-use planning matrix for flood risk reduction.

With respect to the above, a land-use planning matrix was created (Figure 7). The matrix approach, adopted from the Hawkesbury Nepean Flood Management Advisory Committee’s report “*Land-use Planning and Development Control Measures*” [35], allows highlighting the different vulnerabilities of land cover classes to flood hazard. The matrix responds to flood hazard levels through spatial allocation of land-uses. In this vein, the land-use planning matrix is created by demarcating zones by level of flood hazard (low, moderate, and high) and associating them to appropriate, secure, and authorized land-uses:

- High exposure areas are assigned to low-occupancy uses such as recreational activities, ecosystem-based livelihoods involving agriculture, or ecotourism riparian activities. In these areas most development is restricted, and existing development must be prioritized for protection and retrofitting.
- In moderate exposure areas, a “living with water” approach should be adopted through development controls, flood-resistant building codes, and green infrastructure (where possible) for decreasing impermeable surfaces and ameliorating the connectivity between green spaces.
- In low exposure areas, preventive relocation and urban growth is possible with development controls, including strictly enforced building codes, mandatory flood insurance programs, etc.

Urban classes: Targeting built areas (grey infrastructures) is a complex approach. Therefore, the proposed zonation should be coupled to an enforced regulatory framework for halting residential intensification in highly and moderately exposed areas. Construction permits should be reduced, and real estate development for residential purposes should be limited [36]. Building codes should be imposed to indicate minimum design standards for floor levels, materials, and access points. Moreover, if damaged, these artificial areas are not recommended to be repaired but to be relocated and replaced by natural cover. such as forests or grasslands. 

Urban sprawls must be controlled during planning phases since they are still in early development and should not be allowed to be evolve into denser classes. It is also important to emphasize that currently urban sprawls are preset as fragments in the landscape and are dispersed in the floodplain. This pattern reveals potential isolation and hence a higher vulnerability. The exposure of already existing buildings cannot be modified. However, their vulnerabilities can be reduced through retrofitting measures.

On the other hand, in urban cover destinated for recreation (restaurants, public parks, sport facilities, etc.), the presence of people should be regulated not to extend beyond overnight stays, particularly in highly exposed areas. Open space/recreational areas may also be utilized to maximize water retention capacities. For this purpose, these should be designed to function with a dual purpose. 

Al-Assi restaurants, scattered along the river in highly exposed areas and representing a well-known destination in Lebanon, can be preserved. However, the evacuation of visitors and workers should be well planned for in the case of flooding events. Concerning the road network, it should be strategically located and built with appropriate heights to preserve emergency routes “to and within” flood zones. Establishment of public facilities, such as educational facilities, hospitals, commercial centers, etc. should not be allowed in the floodplain. The development of structural protection (e.g., levees, dams, etc.) should be done carefully to avoid their unwanted side-effects. Here, the grey (hard infrastructure) versus green (natural solutions) debate is an open-ended question. Usually, structural protection suppresses the hazard area and opens its land to development, increasing associated exposures. According to the Australian Institute for Disaster Resilience (2018), structural protection systems incentives and feedbacks encourage risk accumulation [37]. Furthermore, if not properly maintained due to lack of resources or simply negligence, these protective structures may collapse and cause cascading catastrophes [38]. Accordingly, careful planning of flood mitigation infrastructure (whether green, grey or hybrid) is a must. Finally, obstruction of river flows should be totally prohibited.

Agricultural zones: In terms of agriculture, the preservation of existing agricultural cover is a must. Agricultural areas are usually the most severely impacted by floods. Flood adaptation measures include farming restriction to non-flood periods, cultivation of flood-resistant species, and the construction of levees, private dikes, and stock refuges. For more sustainable approaches, the use of agricultural Nature-Based Solutions [39] such as agorecology or agroforesty is a very interesting platform to consider. Nevertheless, the advantages should be weighed against the loss of nutrient replenishment from the river and the potentially increasing river erosion. However, some advantages from flooding could be agricultural expansion during post-flooding periods where fertile alluvial soils are deposited. 

Natural classes: Promoting the development natural cover (forest, grasslands, scrublands, etc.), through ecological protection and restoration [40], is of considerable importance to reduce flood hazard. It is highly recommended to preserve at least 80% of lands with gardens, orchards, grass, or vegetables (permeable surfaces). The specific goal of this zonation is to create a buffer zone for enhancing water retention and infiltration, reducing soil erosion and landslides risks while providing green spaces, parks, and recreational amenities to Al-Assi floodplain community. Fish-breeding activities are well-spread throughout the riverbanks. Already in place, riparian systems must be protected for ensuring river banks’ integrity, water quality, ecological connectivity, and viable habitats for biodiversity. Natural areas do not only reduce floods, but also improve the communities’ livelihoods through ecosystem services and recreation (outdoor sports facilities, parks, nature reserves). Buffer zones were implemented in the U.S.A. from the 1970s. Per example, Americans are aware that the loss of wetlands increases storm surge hazard along the Gulf Coast, and specifically in the Mississippi Delta. Several studies have reached similar conclusions for inland floodplains. In this vein, the compensation principle for wetland losses under Section 404 of the Clean Water Act of 1972 [41] was introduced. Moreover, strategic natural spaces can be used for emergency and relief operations as locations for temporary shelters and medical field stations.

Unproductive areas: These areas must be converted into natural classes where possible, by taking into consideration slopes and the underlying’s soil properties. Those suitable for productivity must be optimized, those on low hazards must be planted, while those on moderate and high hazard levels must be overlaid by vegetative protective cover. This reduces the fraction of exposed soils to floods and can assist in flood mitigation. 

### 3.3. Application, Advantages, and Limitations of the Approach

The proposed zoning ordinance should be implemented at the local/municipal level, since the municipality grants building permits, and is monitored by the governorate and national subdivisions. The success of implementing the proposed land-use planning, lies in the fact that the proposed measures are less costly than structural measures. They are also easy applicable during recovery phases for communities, such as those of Al-Assi floodplain and Baalbek-Hermel. They can also assist in the allocation of adequate measures in cases where budgets are limited or scarce. Moreover, living in a floodplain with risk-informed land use planning would necessarily raise awareness among inhabitants.

However, this approach faces a number of constraints, especially in the case of Al-Assi floodplain, and other regions of developing countries [42]. These encountered gaps, challenges, and uncertainties fall under the following categories: uncertainties due to climate change, unclear tenure and contentious land ownership, dynamic changes leading to rapidly outdated plans, societal unacceptance/locals consent, difficulty of relocation, lack of laws and flood insurance, political and legal implications, and jurisdictional/risk borders.

#### 3.3.1. Climate Change

The proposed matrix was based on flood hazards without taking into consideration climate change. However, the latter amplifies flood hazards and their damage, particularly in developing countries [43,44]. With climate change, flood depth may become higher and the extent of the floodplain may become greater. For more efficient DRR measures, land use planning must accommodate uncertainties by incorporating the effect of climate change to design more resistant and sustainable approaches. In this vein, the Delphi approach, a well-established and widely used forecasting process framework, can be used to analyze and predict climate change impacts in the concerned floodplains [45,46]. 

#### 3.3.2. Continuous Updates

With changes of socioeconomic conditions and the development of new needs, the established land use plan must extend beyond a static nature. The established land use plan must be periodically updated to represent the changing conditions. 

#### 3.3.3. Poor Land Tenureship and Contentious Ownership

Land tenureship is poorly addressed in Lebanon [47]. State classified lands represent only 10% of the country; this state often leads to continuous uncontrolled exploitation of unclassified lands, which correspond to green natural areas [48]. As a result, agricultural and natural spaces are experiencing sharp declines due to uncontrolled urbanization, lack of governance, poor land stewardship, and the absence of management plans [27]. Accordingly, soil protective cover is being removed, hence amplifying flood risks. While zonation can be an efficient solution for this problem [49], Implementation of land zonation in Al-Assi floodplain, as in other regions of a fragile state like Lebanon, is a challenge [47]. This is mainly due to the conflicts over land ownership. Such challenges impede accurate land use planning and considerably hinder proper land use allocation [50]. The development of a digital cadastral database, indicating land boundaries and ownership, could help in the resolution of this issue. Nonetheless, this task is complicated by the presence of informal settlements that are often illegal encroachments over private lands. Accordingly, the current status of lands in Lebanon, as well as the study area, are a major challenge for efficient hazard-informed land use planning. 

#### 3.3.4. Societal Unacceptance/Locals’ Consent

Although the efficiency of land-use planning in decreasing flood risks is generally recognized by decision-makers, according to White et al. (2005) [51], its implementation is locally controverted and even faces reluctance. This gap can be considered as the “Achilles’ tendon” of any non-structural land-use based DRR policy. Adaptation measures that reduce benefits from economic assets and restrain land use rights are highly unpopular. This leads to biases in governments’ DRR actions. Locals prefer to see structural measures that ensure that flood risks are being addressed. In fact, locals would have difficulties to understand the costs versus benefits of spatial planning, specifically when comparing green infrastructure to conventional measures. Furthermore, risks predicted by scientists may not be perceived as “real” by the community. Any overall plan would also require trade-offs in implementation because of stakeholder participation. The participatory method, as defended by Ikeda et al. (2008) [52] and Rouillard et al. (2014) [53], is believed to help the societal acceptance of land-use planning.

#### 3.3.5. Relocation and Resettlements

No person would accept to be resettled out of their land or would tolerate to see their lands marked as unsuitable for construction or investment [54]. Preventive resettlement and relocation of residents is chosen as a last resort for creating flood retention zones. Such an approach is very difficult in Al-Assi floodplain and in Lebanon. This difficulty derives from the scarcity of lands due to excessive encroachment on lands [55], and a lack of consensus or approval among locals. Moreover, zoning affected areas can become politicized and relocations can be regarded as efforts to reattribute lands to more powerful interests [24]. Lack of resources also represents an important impediment in the application of land use zoning [56]. Governments should provide alternative safe lands instead of those where construction and investment are banned. 

#### 3.3.6. Lack of Flood Insurance

Many developing countries such as Lebanon do not have disaster-related insurance measures [57]. The Lebanese Government is not capable of compensating all losses. Flood insurance imposing high rates on restricted lands constitutes an important support to land-use planning adoption and also plays a significant role in flood risk communication [58]. Some insurance schemes may be imposed to encourage self-protection by decreasing premium amounts or increasing pay-outs.

#### 3.3.7. Political and Legal Implications

To be efficient, land-use planning needs to be accompanied by legislations and jurisdictional-based approaches; “The regulatory framework of non-structural measures is the key for ensuring that actors carry out their roles and responsibilities with the full information and the right incentives” [22]. Legal procedures must be initiated, and serious penalties must be imposed if constructions and land cover expansion do not fit into the land use ordinance matrix. In Lebanon, lack of laws related to this issue, the long time needed to issue such laws, and the numerous political involvements would hamper the implementation of land zoning decisions.

#### 3.3.8. Jurisdictional and Risk Borders

Hazard and risk do not overlap with the administrative borders, and their mitigation constitutes a cross-jurisdictional issue. Flood risk can be shared by more than one administrative level—three villages in the case of Al-Assi floodplain (Hermel, Zighrine, and Deir Mar Maroun Baalbek) are implicated. The involvement of multiple municipalities may constitute a challenge to the implementation of land-use zoning ordinance and planning due to lack of collaboration, coordination, and cooperation. A review of risk governance scales is a must.

Finally, had values of the flood at various stretches of the river been available, clearer insights on the magnitude of floods would have been provided. Such considerations could indicate to what extent and by what magnitude floods could be addressed by structural or non-structural measures. While these values and measurements were missing in this study, their integration where available is highly recommended for a more holistic approach.

## 4. Conclusions

This paper highlighted the role of land use planning in boosting DRR governance, especially in developing countries. This approach was done by discussing DRR measures from the perspective of flood-risk mitigation and highlighting the importance of spatial planning for better land use planning. The evaluation of the LU/LC timeseries of Al-Assi floodplain revealed that although Al-Assi River has flooded several times in the past and caused tremendous damage and losses, no land-use planning has been carried out. Even recovery efforts were often unplanned and led to the reconstruction of the same structures at the same locations. A rate of +6.15% of urban cover was found. Such an evolution is remarkable in a floodplain. In an attempt to improve this alarming situation, a hazard-informed LU/LC planning matrix was developed. This matrix demarcates areas by levels of flood hazard (low, moderate, and high) and links them to appropriate, safe, and permissible land uses/controls where necessary. The proposed land use planning measures focused on minimizing development in the floodplain, reducing water runoff through development controls for flood risk mitigation, mitigating damages from unavoidable floods, and accommodating urban growth and expansion in flood-safe areas (resettlement and reconstruction). This scientifically-based land use planning serves as a decision-making and risk communication support tool. It is believed that the outcome of this study would help in convincing municipalities and locals to participate in the implementation of the measures, communicate risks, spread the culture of “living with risk”, and strengthen social cohesion. Living with floods through land-use planning is believed to increase public awareness and, ultimately, resilience. However, some constraints, specific to developing countries, can hinder the adoption and implementation of the proposed land-use planning. Through use the use of satellite data, the methods proposed in this study can be easily replicated, while the challenges listed can serve as further developments of this approach in contexts similar to the Assi floodplain.

## Figures and Tables

**Table 1:** Utilized multispectral satellite imageries for LULC mapping.

Satellite	Date of Acquisition	Pan Sharpened Spatial Resolution (m)
Landsat and IRS	1998	5.8
IKONOS	2005	1
GEOEYE	2013	0.5
SPOT	2018	1.5

**Table 2:** LULC time series analysis results.

		Area (km^2^)				Percentage Change				Rate of Change (%/Year)
LU/LC Classes		1998	2005	2013	2018	1998–2005	2005–2013	2013–2018	1998–2018	1998–2018
**Forest and semi natural areas**	Clear-other types of Broadleaved trees	0.002	0.06	0.005	0.005	+0.99	−0.93	0	0.003	0.00015
	Clear Juniper		0.64	0.35	0.35	+10.21	−4.7	0	0.35	0.0175
	Clear Mixed Wooded Lands	0.002	0.30	0.24	0.24	+4.78	−1.06	0	0.238	0.0119
	Clear Oaks	-	-	0.03	0.03	0	0	+0.42	0.03	0.0015
	Scrubland	0.69	-	0.004	0.004	−10.1	+0.06	+0.004	−0.686	−0.0343
	Scrubland with Some Dispersed Bigger Trees	0.15	0.12	0.008	0.008	−0.46	−1.83	0	−0.142	−0.0071
	Medium density grasslands	0.07	-	-	-	−1.18	0	0	−0.07	−0.0035
	Clear Grasslands	2.18	0.16	0.06	0.06	−32.22	−1.5	−0.14	−2.12	−0.106
	**Total**									**−2.397**
**Agricultural Areas**	Field Crops in Medium to Large Terrace	0.33	0.44	2.06	1.98	+1.75	+25.79	+1.32	1.65	0.0825
	Field Crops in Small Fields/Terrace	0.19	1.46	0.40	0.43	+20.13	−16.84	+0.57	0.24	0.012
	Fruit Trees	0.07	0.69	0.41	0.49	+9.85	−4.49	+1.31	0.42	0.021
	Olives	-	0.0003	0.11	0.11	+0.006	+1.72	−0.03	0.11	0.0055
	Protected Agriculture	1.26	-	0.009	0.009	−19.97	+0.15	0	−1.251	−0.06255
	**Total**									**+1.169**
**Water Bodies**	Hill Lake	-	0.006	0.03	0.03	+0.10	+0.37	0	0.03	0.0015
	Lake	-	-	0.001	0.001	0	+0.02	0	0.001	0.00005
	**Total**									**+0.031**
**Artificial Surfaces**	Recreation	-	0.01	0.02	0.05	+0.23	+0.06	+0.4	0.05	0.0025
	Low Density Urban Fabric	0.05	0.05	0.19	0.26	+0.02	+2.27	+1.07	0.21	0.0105
	Medium Density Urban Fabric	-	-	-	0.000032	0	0	+0.0005	0.000032	0.0000016
	Urban Sprawl on Clear Wooded Land	-	0.02	0.04	0.035	+0.30	+0.26	0	0.035	0.00175
	Urban Sprawl on Field Crops	-	0.01	0.02	0.04	+0.23	+0.06	+0.33	0.04	0.002
	Urban Sprawl on Permanent Crops	-	-	0.008	0.06	0	+0.14	+0.83	0.06	0.003
	Tourist Resort	-	0.04	0.06	0.02	+0.56	+0.41	−0.69	0.02	0.001
	**Total**									**+0.415**

**Table 3:** Current LULC classes exposure level to flood hazard.

	Flood Hazard Level		
LU/LC 2018	Low	Moderate	High
Unproductive areas	X	X	X
Diverse Equipment	X	X	
Mineral Extraction Site	X		X
Clear-other types of Broadleaved trees	X	X	X
Clear Juniper	X	X	
Clear Mixed Wooded Lands	X	X	X
Clear Oaks	X	X	X
Scrubland	X	X	
Scrubland with Some Dispersed Bigger Trees	X	X	
Medium density grasslands	X	X	X
Clear Grasslands	X	X	X
Field Crops in Medium to Large Terrace	X	X	
Field Crops in Small Fields/Terrace	X	X	X
Fruit Trees	X	X	X
Olives	X	X	X
Protected Agriculture	X	X	X
Recreation		X	X
Hill Lake		X	X
Lake		X	
Low Density Urban Fabric	X	X	X
Medium Density Urban Fabric	X		
Urban Sprawl on Clear Wooded Land	X	X	X
Urban Sprawl on Field Crops	X	X	X
Urban Sprawl on Permanent Crops	X	X	X
Tourist Resort	X	X	X
Industry		X	

X LULC class presence. X Misplaced LULC class.

## Data Availability

The data presented in this study are available on request from the corresponding author.
